# Comparison of Cardiopulmonary Resuscitation Quality in a Simulated Model: At Incident Scene vs During EMS Transport

**DOI:** 10.5811/westjem.40234

**Published:** 2025-09-25

**Authors:** Murat Çetin, Gökhan Yilmaz, İlhan Uz, Turgay Yılmaz Kılıç, Erkan Guvenç, Volkan Ergun, Ebru Şener Araz, Başak Bayram, Brit Jeffrey Long, Michael Gottlieb, William J. Brady

**Affiliations:** * Department of Emergency Medicine, Izmir Dr. Behçet Uz Children’s Disease and Surgery Education and Research Hospital, Izmir, Türkiye; † Department of Emergency Medicine, Konya Meram Public Hospital, Konya, Türkiye; ‡ Department of Emergency Medicine, Faculty of Medicine, Ege University, Izmir, Türkiye; § Department of Emergency Medicine, Izmir Faculty of Medicine, University of Health Sciences, Izmir Bayraklı City Hospital, İzmir, Türkiye; ¶ Izmir Metropolitan Municipality Esrefpasa Hospital, Izmir, Türkiye; || Department of Emergency Health Services, Izmir Provincial Directorate of Health, Izmir, Türkiye; # Department of Emergency Health Services Training Unit, Izmir Provincial Directorate of Health, Izmir, Türkiye; ** Department of Emergency Medicine, Brooke Army Medical Center, Fort Sam Houston, TX, USA; †† Department of Emergency Medicine, Rush University Medical Center, Chicago, IL, USA; ‡‡ Department of Emergency Medicine, University of Virginia School of Medicine, Charlottesville, VA, USA

## Abstract

**Introduction:**

Out-of-hospital cardiac arrest remains a leading cause of death and significantly impacts global health outcomes. International guidelines emphasize the importance of high-quality CPR (cardiopulmonary resuscitation).

**Objectives:**

Our goal was to compare CPR efficiency using the criteria recommended by international guidelines between two out-of-hospital cardiac arrest intervention scenarios: CPR at the incident site; and CPR during patient transport to the hospital by emergency medical services.

**Methods:**

In each of the two scenarios, five full two-minute cycles of cardiac compression were applied to a manikin according to international guidelines. The CPR quality parameters were chest compression rate, chest compression depth recorded by the manikin, and investigator-evaluated correct hand placement on the manikin.

**Results:**

We analyzed data from 240 CPR cycles provided by 24 healthcare professionals. The mean chest compression rate was higher (120.5±10.9/minutes vs 125.3±14.7/min, P = .001) and the mean chest compression depth was shallower (43.9±6.6 millimeters [mm] vs 37.9±7.2 mm, P = .001) in the on-the-move group. The two groups’ appropriate hand placement rates were similar (92.1±5.4% vs 92.2±4.5%, P = .48)

**Conclusion:**

In this study, the moving ambulance simulation demonstrated that chest compressions were administered at a rate exceeding recommended guidelines and at a shallower depth than recommended, while the frequency of correct hand placement remained comparable. If the patient requires transportation from the scene of the incident, the healthcare team must be aware of the potential adverse effects on the chest compression quality.

## INTRODUCTION

Out-of-hospital cardiac arrest (OHCA) remains a leading cause of death and significantly impacts global health outcomes.^1^ Early recognition, early cardiopulmonary resuscitation (CPR), and defibrillation are the key components of the first response to immediately restore and maintain circulation, as emphasized in the CPR guidelines by major health authorities such as the American Heart Association (AHA) and the European Resuscitation Council (ERC).^2^,^3^ These guidelines emphasize the importance of high-quality CPR. Key components of high-quality CPR include maintaining an adequate chest compression rate of 100–120 compressions per minute and a chest compression depth of 5–6 centimeters (cm), allowing for full chest recoil and minimizing interruptions.^4^ Despite the critical nature of high-quality CPR, various factors can compromise its effectiveness, including human fatigue and logistical challenges during patient transport.

Performing effective, high-quality CPR is crucial for survival and prevention of morbidities in OHCA.^5^–^8^ However, previous studies revealed inadequate CPR efficiency and duration during the intervention and transportation of OHCA patients to healthcare facilities.^9^–^11^ Several more recent studies have demonstrated better CPR quality during the transport of OHCA cases.^12^ However, there have been variations in methodology prompting the need for studies in controlled settings with easily reproducible methods.

The decision to perform CPR on site or during transport in OHCA cases remains a significant challenge for prehospital healthcare responders. Various factors, including patient-related factors, local circumstances, paramedic-related factors, and the organizational structure, can influence this decision.^13^ However, one of the most important questions is whether effective CPR can be performed in a moving ambulance. In this study we aimed to compare the efficiency of CPR performed on site vs during transport, using the latest criteria recommended by current CPR guidelines.

## METHODS

### Study Setting and Design

This manikin-based practice assessment study was conducted on 3–4 November 2022 to evaluate the compression quality comparatively between two settings common to OHCA incidents. Participants applied a standardized chest compression methodology recommended by AHA and ERC guidelines, with two minutes of chest compression cycles followed by 10 seconds of pulse check in both scenarios. No real-time feedback devices were used during CPR. The on-site scenario involved performing CPR on the scene of the OHCA event, while the on-the-move scenario involved beginning CPR while taking the manikin off the cot, continuing while loading it into the ambulance, and transporting it to the hospital.

Hypothetically, the OHCA scene was deemed a safe roadside location 10 kilometers (km) from the hospital point. In both scenarios, the healthcare team departed from the hospital and arrived by ambulance at the scene of the incident. While the on-site scenario was managed entirely at the scene, the on-the-move scenario involved transport from the scene of the event to the hospital with compression continued in the ambulance, returning to the hospital point at a 50 km/hour speed on the road with two bends and two bumps on the route. Each scenario was conducted for five cycles. Healthcare team members were selected from volunteer emergency medical technicians (EMT) with < two years of professional experience. Participants were not informed of the study hypothesis. Volunteers were excluded if they had conditions that might affect their CPR practice (eg, motion sickness, dizziness).

**Population Health Research Capsule**

What do we already know about this issue?

*High-quality cardiopulmonary resuscitation (CPR) is essential for survival in out-of-hospital cardiac arrest (OHCA). However, CPR quality can be compromised by factors such as rescuer fatigue and patient transport conditions*.

What was the research question?

*Does the quality of CPR performed during ambulance transport differ from that performed at the incident scene?*

What was the major finding of the study?

*Chest compressions delivered in a moving ambulance were performed at a higher rate but were significantly shallower compared to those administered on site, while hand placement accuracy remained similar*.

How does this improve population health?

*Understanding the limitations of CPR quality during transport emphasizes the importance of on-site resuscitation, targeted training, and system-level planning. This knowledge can improve outcomes in OHCA by guiding EMS strategies to maximize CPR effectiveness before and during transport*.

We evaluated CPR efficiency by measuring the chest compression rate (compression per minute) and chest compression depth (millimeters) recorded by the manikin during CPR and the appropriateness of hand placement on the manikin (correct/incorrect) per the evaluator’s judgment. We calculated the average values of the chest compression rate and depth measurements and the percentage of correct hand placements from five cycles and recorded the values as the assessment result for each participant. The institutional review board at Necmettin Erbakan University, Konya, Türkiye (2022/4047), approved this study.

### Statistical Analysis

After controlling for normal distribution assumptions, we presented the continuous data using mean score and 95% Cl for normally distributed variables. Categorical data were presented using frequency and percentage values. We compared continuous data using the independent samples t-test between independent groups and the paired-samples t-test between dependent groups. A type 1 error level of 5% was considered the statistically significant threshold in a 95% CI. A sample size estimation based on the two related groups designed to detect a difference of 0.7±1 cm difference for chest compression. Depth between the two scenarios with 80% of study power revealed that 19 participants were needed as the study population. With the addition of a 20% attrition margin, we recruited 24 participants for the study. All statistical analyses were done in SPSS 22 (IBM Corporation, Armonk, NY).

## RESULTS

Twenty-four participants, with a mean age of 23.9 years of age (SD 1.7 years) and 18 of whom were males (75%), completed 240 cycles of CPR. The aggregated and per-cycle results for CPR assessments in the on-site and on-the-move groups are presented in Table 1 The mean chest compression rate was higher (120.5±10.9 /min vs 125.3 ± 14.7 /min) and the mean chest compression depth was shallower (43.9 ± 6.6 mm vs. 37.9 ± 7.2 mm) in the on-the-move group. The two groups’ appropriate hand placement rates were similar.

We also compared the CPR assessment criteria measurements between the first and last cycles (Table 2). The chest compression rate measurements were similar in both groups. The chest compression depth measurements were significantly higher in the first cycle in both the on-site (49.2 ± 7.5 mm vs 39.7 ± 6.5 mm) and the on-the-move groups (41.8 ± 8.4 mm vs 34.7 ± 8.5 mm). However, appropriate hand placement rates were higher in the last cycles of both groups (on site: 85.9 ± 9.8% vs 93.5 ± 3.7%; on the move: 85.8 ± 7.6% vs. 93.5 ± 4.3%).

## DISCUSSION

A key component of OHCA cardiac arrest management is maintaining high-quality CPR. In this study we found that on-the-move CPR resulted in higher chest compression rates and shallower chest compression depths. Not maintaining an optimal chest compression rate is associated with inadequate recoil and ventricular filling, whereas non-optimal chest compression depth may result in inadequate cardiac output and perfusion. The previous evidence suggests that poor-quality CPR might affect return of spontaneous circulation since an appropriate chest compression rate is needed to establish an optimal coronary blood flow to achieve spontaneous circulation.^14^

One factor affecting the differences between settings might be the relationship between the chest compression rate and depth. A previous CPR-focused manikin study showed that participants were unable to perform CPR at sufficient depth as their rate of administering chest compressions increased.^15^ Accordingly, chest compression depth could not be achieved in > 50% of applications when the chest compression rate was 120/min and in > 60% when the rate was 130/min. Our study supports the interconnectedness of these findings. Another study by Havel et al^16^ reported that the chest compression depth was lower than optimal on the scene and in the moving ambulance (the scene group 3.7 cm vs the moving ambulance group 3.6 cm). Our chest compression depth results in the on-the-move group were similar to those reported in the study by Havel et al, but our on-site group had higher chest compression depth than the reported values.

Based on these previous findings, we can postulate that EMTs might have felt more stressed in the moving ambulance and could not apply adequate pressure for a sufficient chest compression depth. This may also explain the higher chest compression rate, which eventually caused fatigue and further decreased the chest compression depth. Other potential factors could be the narrow range of motion in the ambulance, environmental problems like bumps and bend in the road, and other suboptimal conditions caused by the movement of the ambulance, which may complicate CPR and prevent the first responder from performing CPR at sufficient depth.

**Figure 1:**
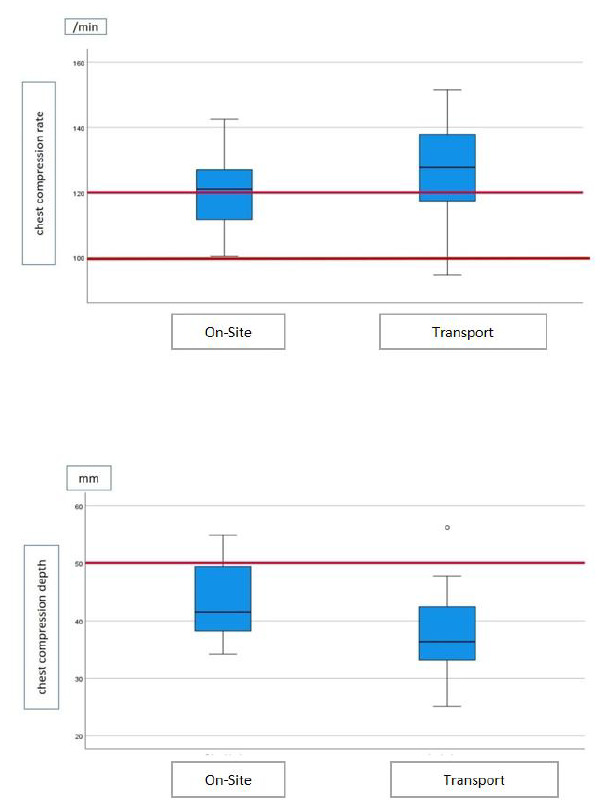
Status of the quality values of cardiopulmonary resuscitation performed by first responders in our study, compared to standard CPR. *CPR*, cardiopulmonary resuscitation; *min*, minute; *mm*, millimeter.

In our study, chest compression rate was similar between the first and last cycles in both scenarios. However, EMTs performed CPR closer to the recommended depth at the first cycle on site and below the recommended depth in the moving ambulance (Figure 1). In addition, they failed to achieve adequate chest compression depth through the later cycles. Bjorshol et al^17^ reported that only half of the subjects who completed chest compressions performed CPR at the desired quality, that the quality of CPR did not deteriorate in the first two minutes of CPR, and the CPR quality deteriorated due to fatigue in the CPR practitioner toward the end of CPR. Hightower et al^18^ also evaluated the temporal variation of chest compression quality and asked the subjects to perform CPR for five minutes; they found that the average compression efficiency decreased by 90% after the first minute. In the same study, the chest compression rate was preserved, but the chest compression quality decreased over time. Swapping the person performing chest compressions could potentially be an effective way to counteract fatigue. On the other hand, the hand placement in both scenarios improved over time. This might be related to the lack of experience of the EMTs using the manikin and implies the need for dedicated time to get used to the manikin to eliminate the initial stress.

The results of our study suggest that on-the-move CPR is more prone to decreased quality due to shallower chest compression depth and higher rate of chest compressions, both of which might affect the outcomes in OHCA management. Some steps to be taken to avoid the negative consequences of OHCA management can include the following: applying all CPR on site until the patient is stabilized for transport; more focused training for on-the-move CPR; and monitoring of on-the-move CPR with directed feedback. Since the evidence in the literature varies between studies and settings, there is a need for a more standardized protocol to evaluate ambulatory EMT applications for OHCA management, which might also be used for internal assessment and training of these first responders.

## LIMITATIONS

Several limitations should be considered for this study. First, this was a manikin-based simulation study, which might not truly reflect the practice patterns in actual OHCA incidents. Second, because this study was conducted at a single site, the participants’ CPR performance may not be generalizable to all EMS personnel. In this study, we evaluated the CPR practices of EMTs with < two years of experience. It can be anticipated that the results might differ with more experienced healthcare professionals. Third, as the goal of CPR is the return of spontaneous circulation, our manikin simulation could not detect this outcome; however, given the emphasis in the existing literature on high-quality CPR as a key link in the chain of survival, we believe these findings remain highly relevant to patient care.

Lastly, we did not evaluate rebound time and compression fraction—important indicators of CPR quality—due to limitations of the manikin used in the study. Additionally, the absence of real-time feedback tools, such as a metronome, feedback device, or end-tidal carbon dioxide monitoring, during both on-scene and on-the-move CPR represents another limitation. This lack of feedback may have affected the consistency and quality of CPR performance, potentially influencing the study outcomes. Regarding the use of mechanical chest compression devices, we believe that such equipment can offer consistent compression quality, especially in challenging environments. However, in many low- and middle-income countries, including our own, access to these devices is limited due to financial and logistical constraints. As highlighted in recent literature, the scarcity of essential medical equipment, including mechanical CPR devices, remains a significant barrier in resource-limited settings.

Besides these limitations, our study provides the most current data for local applications in Türkiye for OHCA management, according to the most recent guidelines, and highlights areas to improve the administration of CPR by first responders.

## CONCLUSION

In this simulated model, performing CPR on the move resulted in a small increase in chest compression rate and significantly shallower chest compression depth compared with on site. Based upon these findings, we recommend performing CPR on site when possible. Future studies evaluating CPR quality in actual patients, comparing the effects of on-site vs transport CPR, would be valuable in characterizing the quality of resuscitation for out-of-hospital coronary arrest.

## Figures and Tables

**Table 1:** Aggregated and per-cycle cardiopulmonary resuscitation assessment in study groups

	On site		On the move	
	
	Mean Score	%95 CI	Mean Score	%95 CI
Chest compression rate (compressions/min)				
1st cycle	123.4	117.8–129	125.2	119.1–131.4
2nd cycle	120.0	115.2–124.8	125.7	118.9–132.3
3rd cycle	120.6	115.9–125.3	125.6	118.9–132.3
4th cycle	120.3	115–125.7	125.6	119.1–132.1
5th cycle	118.4	113–123.8	124.5	117.7–131.4
Average	120.5	116–125.1	125.3	119.1–131.4
Chest compression depth (mm)				
1st cycle	49.2	46–52.3	41.8	38.2–45.3
2nd cycle	45.6	42–49.2	38.7	35.7–41.7
3rd cycle	43.4	40.3–46.5	37.7	34.6–40.8
4th cycle	41.8	38.9–44.7	36.4	32.8–40
5th cycle	39.7	36.9–42.4	34.7	31.1–38.2
Average	43.9	41.1–46.7	37.9	34.8–40.9
Appropriate hand placement (%)				
1st cycle	85.9	81.8–90.1	85.8	82.6–89
2nd cycle	91.8	88.7–94.8	92.6	90.5–94.8
3rd cycle	94.3	92.1–96.4	94.1	91.9–96.3
4th cycle	95.0	93.4–96.6	94.8	92.9–96.8
5th cycle	93.5	91.9–95	93.5	91.6–95.3
Average	92.1	89.8–94.3	92.2	90.3–94.1

/*min*, per minute; *mm*, millimeter.

**Table 2:** Comparison of cardiopulmonary resuscitation assessments between the first and last of five cycles.

	On site				On the move			
	
	1st cycle		5th cycle		1st cycle		5th cycle	
	
	Mean Score	%95 CI	Mean Score	%95 CI	Mean Score	%95 CI	Mean Score	%95 CI
Chest compression rate (/min)	123.4	117.8–129	118.4	113–123.8	125.2	119.1–131.4	124.5	117.7–131.4
Chest compression depth (mm)	49.2	46–52.3	39.7	36.9–42.4	41.8	38.9–44.7	34.7	31.1–38.2
Appropriate hand placement (%)	85.9	81.8–90.1	93.5	91.9–95	85.8	82.6–89	93.5	91.6–95.3

*/min*, per minute; *mm*, millimeter.
